# Children's perceptions of physical literacy: exploring meaning, value, and capabilities for lifelong physical activity

**DOI:** 10.3389/fspor.2025.1548546

**Published:** 2025-05-14

**Authors:** Daniel D. Bingham, Jade L. Morris, Kiara Lewis, Lawrence Foweather, Hannah Goss, Wesley O’Brien, Inimfon Essiet, William M. Roberts, Liezel Hurter, Cara Shearer, Lisa M. Barnett, Michael J. Duncan, Andrew Daly-Smith

**Affiliations:** ^1^ Faculty of Health Studies, University of Bradford, Bradford, United Kingdom; ^2^ Centre for Life and Sport Sciences, Birmingham City University, Birmingham, United Kingdom; ^3^ Research Institute for Sport and Exercise Sciences, Liverpool John Moores University, Liverpool, United Kingdom; ^4^ School of Health and Human Performance, Dublin City University, Dublin, Ireland; ^5^ Physical Education, Sports Studies and Arts Programme, School of Education, University College Cork, Cork, Ireland; ^6^ School of Sport and Human Movement, University of Waikato, Hamilton, New Zealand; ^7^ Institute of Physical Activity and Nutrition, Deakin University, Melbourne, VIC, Australia; ^8^ Centre for Physical Activity, Sport and Exercise Science, Coventry University, Coventry, United Kingdom

**Keywords:** physical activity, physical literacy, children, young people, qualitative

## Abstract

**Introduction:**

The concept of physical literacy has been defined differently across the world. To create a consensus statement and definition of physical literacy for England, it was felt important to incorporate the views and opinions of children and young people who are often the focus of interventions to increase physical activity and physical literacy. The aim of this qualitative study was to understand what physical literacy means to children by exploring their perceptions of meaningful physical activity and what they think will be needed to continue to be active for life.

**Methods:**

Through a series of directed tasks and thematic analysis, several important considerations are discussed. These included the pertinence of social relationships-whether it was to share experiences, support and encourage friends, or learn from your family.

**Results and Discussion:**

Children discussed how physical activity positively affects their emotions and the importance of enjoyment in continuing to engage in movement for the rest of their lives. In addition, there was an awareness of the benefits for mental and physical health, which indicated the prominence of knowing these benefits in engaging. The findings offer some important contributions from children to better understand what physical literacy means in England.

## Background

Physical literacy (PL) is frequently described as a holistic concept that extends beyond physical activity (PA) alone. While PA refers to any bodily movement produced by skeletal muscles that requires energy expenditure (1), PL extends this focus by encompassing the skills, knowledge, attitudes, and motivation needed for confident and continuous engagement in various forms of movement and physical activities throughout life (2). At its core, PL seeks to foster a lifelong relationship with movement by integrating physical, cognitive, affective, social, and even spiritual domains (3, 4), distinguishing PL from more behaviour-centric concepts of PA. Physical education (PE) plays a fundamental role in introducing children to structured movement and physical activity experiences that contribute to their physical and psychosocial development, improving motor skills, cognitive function, social competence, and fostering engagement in lifelong activity (5). However, participation in PE varies widely, with some children disengaging due to factors such as lack of enjoyment, perceived incompetence, or negative social experiences. Given these disparities, it is critical to explore how children themselves perceive meaningful movement experiences beyond structured PE settings. This study, therefore, focuses on children and young people's (CYP) perceptions of PL to understand how they conceptualise movement holistically and what factors contribute to lifelong physical activity engagement across multiple contexts.

Over the past 10 years, PL has garnered increasing attention in research, policy, and practice (1, 6–8). Contributing to sustainable development goals by promoting wellbeing and population health (6, 9), PL is often framed as an alternative or complementary approach to standard PA recommendations. Whitehead (2) originally conceptualised PL as an embodied, holistic notion, emphasising the mind–body connection. This perspective suggests that engaging in enjoyable, meaningful, and personally valued forms of movement can deepen one's connection to activity and encourage an ongoing commitment to an active lifestyle (10). Despite this potential, inconsistent definitions of PL have emerged worldwide (8, 11–13). Some definitions see PL as primarily concerned with physical and motor competence (12), thereby narrowing its holistic scope (13). In contrast, contemporary discussions highlight broader elements such as motivation, confidence, and understanding (11, 14), as well as social and spiritual domains (4). These varied interpretations pose challenges for translating PL into research, policy, and practice (15–17). In response, several countries have initiated the development of PL consensus statements, often via expert panels, Delphi methods, and multi-stakeholder engagement (3, 16–18). While a single universal definition of PL may be neither possible nor universally desirable (2), establishing a shared language fosters alignment among organisations at different levels (19).

Most recently, Sport England launched a consensus-building process to cultivate an English nationally relevant PL definition (10). An integral aim of this effort is to reflect children and young people's perspectives, an area that has been relatively underexplored despite its importance (20, 21). The UN Convention on the Rights of the Child (1992) (22) also underscores that children's views should be taken into account in all matters affecting them; collaborating with children can lead to co-creating definitions that are more meaningful and impactful, while also increasing the agency and leadership of young people themselves; all of which reinforces the need to include young voices in shaping PL frameworks (23–25). While barriers and facilitators to children's PA have been studied extensively (26–29), their intrinsic motivations, values, and core perceptions of PL remain less understood. Martins et al. (11), for instance, in an updated review of qualitative research on adolescents’ activity levels, found that barriers were both physical and psychological, including lack of knowledge and understanding, motivation and perceived competence. Facilitators included having “fun” and access to opportunities, whereas social support was seen as having both a negative and a positive impact, as was the choice of activities. Recent work on the “All Island PL Consensus statement” (30), including a scoping review of existing qualitative research in relation to children, physical education, physical activity, and sport, highlighted the importance of providing choice, promoting fun, positive and negative influences of social influences and competition, and identified potential barriers to positive PL experiences. However, primary data collection of children's perspectives of PL was not possible for the “All Island” consensus work in Ireland due to the COVID-19 pandemic. As a result, research through and with children exploring their perceptions of lifelong physical activity and the physical literacy journey is seen as an important step forward in providing a relevant and contextually understood definition.

Against this backdrop, the aim of this study was to examine children and young people's understanding of PL. The objectives of the study were to (i) understand what children value about participating in movement and physical activity as part of their PL journey and (ii) to understand what PL capabilities/elements children identify as being important for participation in physical activity for life.

## Method

### Recruitment and participants

A purposive convenience sampling approach was employed to recruit schools, driven through school networks and relevant partner networks/organisations with existing relationships with the research team. We sought to recruit schools in the North and South East of England, intending to recruit a diverse sample considering ethnicity, deprivation, and children at special schools with learning difficulties and/or physical disabilities. Ethical approval was provided by the University of Bradford (reference: E989). Following school consent and year group identification, class teachers were asked to identify suitable participants based on pupils with a mix of activity levels (active/sporty vs. inactive/non-sporty) and sex (31, 32). This was to ensure we can capture the opinions and voices of some CYP who regularly participate in movement-based activities and physical activity compared to CYP who have little or no interest or engagement with physical activity and sport.

### Data collection

To encourage divergent perspectives from children on meaningful movement-based activities, we conducted focus groups. Focus groups were conducted in a quiet school space, lasting on average 46 min, ranging from 30 to 58 min. Focus group sizes ranged from three to nine children. Children were asked to complete three tasks that encompassed an activity and a series of questions on meaningful activities involving moving your body (see Supplementary Material S1 for the focus group guide) (33). In this study, “meaningful activities” are informed by Beni et al. (33), who identified five key themes central to meaningful experiences in physical education and youth sport: social interaction, fun, challenge, motor competence, and personally relevant learning. These elements guided the design of the focus group tasks, ensuring they captured a broad range of perspectives and experiences to better understand what children value in movement-based activities.

Task 1 included asking children to draw/write an activity or activities they find meaningful that involve moving their bodies. Children were then prompted to explain the activity in more detail, describing what they were doing, how they felt during and after the activity, and what factors helped them engage in it.

Task 2 required children to think about what is important for maintaining movement and physical activity both now and throughout their lives. Children were encouraged to consider not only their own needs but also those of other children. To support their reflections, prompts were provided to guide their thoughts towards their bodies, minds, environment, and relationships with others. While the task primarily involved writing, some children chose to include drawings as part of their responses.

Task 3, drawing on definitions of PL proposed by researchers (11), aimed to capture children's perspectives on the capabilities or elements critical for engaging in lifelong physical activity. To capture the importance of such elements/concepts from children's perspectives, we drew upon the Australian Physical Literacy Framework (APLF) (34). The APLF includes 30 elements that are housed within four constructs: (i) physical, (ii) psychological, (iii) social, and (iv) cognitive. Each element included a short explanation and example. Examples tailored to Australia were tweaked to provide an example more culturally relevant to England (e.g., building a “den” rather than a cubby). Task three was completed with Key Stage 3 and Key Stage 4 (aged 7–16 years old) children, excluding those with special educational needs and disabilities (SEND). They were asked to rank the 30 elements based on their importance for engaging in movement-based activities both now and in the future. Elements that children found unclear were explained during the task. The exclusion of children with SEND from this task was based on consultation with teaching staff who work with students with SEND, who advised that the cognitive complexity of the ranking process, which required children to compare and prioritise abstract concepts across multiple domains, would likely present significant challenges for many students with additional learning needs. Research supports that such tasks can be particularly difficult for children with cognitive impairments as they require abstract reasoning and complex decision-making (35, 36). While alternative methods, such as verbal discussions or adapted ranking systems, were considered, consultation with teaching staff of participating schools suggested these would not fully address the cognitive demands of the task. Future research should explore inclusive adaptations to ensure greater accessibility in similar ranking-based studies. However, additional adaptations were implemented to ensure accessibility for children with SEND. These included verbal prompts, visual scaffolding, and extended response time, particularly during Tasks 1 and 2. Teachers provided support where necessary, ensuring all children could fully engage in the discussions.

### Data analyses

**Figure 1:**
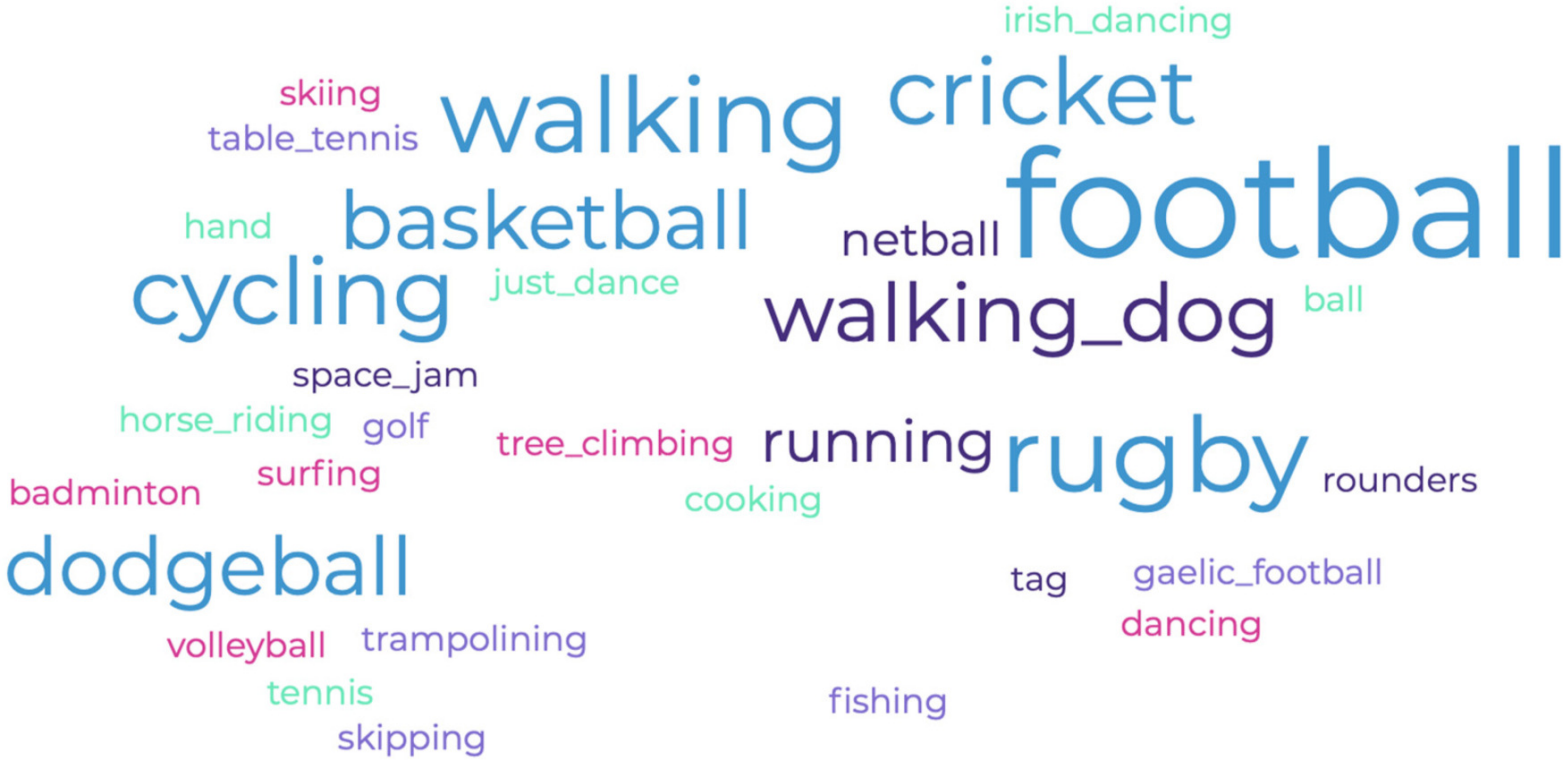
Word cloud of the different movement-based activities the children reported.

The focus groups were audio recorded and transcribed verbatim. Tasks 1 and 2 were analysed based on Hayes’ (37) thematic analysis. First, JM and DB independently coded a subset of transcripts to establish and ensure consistency in theme identification (38). They then compared codes, refining the coding framework through discussion and consensus before proceeding with full dataset analysis. Themes were iteratively developed by grouping similar codes and refining definitions, ensuring they accurately represented children's perspectives. Any themes that overlapped or lacked sufficient supporting data were merged or discarded based on consensus (39). To enhance trustworthiness, regular meetings were held throughout the coding process to discuss discrepancies and refine themes further. The themes are reported below with a selection of supporting quotations as described below. Additionally, a word cloud was produced of the different activities named in task 1 (see Figure 1). Task 3 required children to rank the elements, and, at the same time, recordings and transcription were made of the ongoing discussion. The data are presented as a list of the most highly ranked elements, and some of the discussion themes that arose are presented through the use of quotations.

## Results

A total of 53 participants were recruited for the study (Table 1). Participants were between 10 and 15 years old (M = 13.1 ± 1.8 years). Table 1 demonstrates the distribution of the children’s sex, Key Stage, ethnicity, activity level, and special educational needs and disability.

**Table 1:** Participant characteristics of the recruited sample.

Participant characteristics	*N*	%
Sex		
Girls	22	41.5
Boys	31	58.5
School key stage		
Key stage 2	13	24.5
Key stage 3	23	43.4
Key stage 4	17	32.1
Ethnicity		
White British	35	66.0
Pakistani	9	17.0
Indian	3	5.7
Chinese	2	3.8
Other White	2	3.8
Black	1	1.9
Black and White mixed	1	1.9
Activity category		
Sport/active	31	58.5
Non-sport/inactive	22	41.5
Special educational needs and disabilities		
Autistic spectrum disorder	8	15.1
Learning difficulties	1	1.9
Autistic spectrum disorder and learning difficulties	1	1.9
No special educational needs and disabilities	43	81.1

Key stage 2 includes years 3–6 for pupils aged between 8 and 11 years old. Key stage 3 includes years 7–9 for pupils aged between 11 and 14 years old. Key stage 4 includes years 10–11 for pupils ages between 14 and 16 years old. Special educational needs and disabilities categories were based on school records.

### Activities meaningful to the children (task 1)

**Figure 2:**
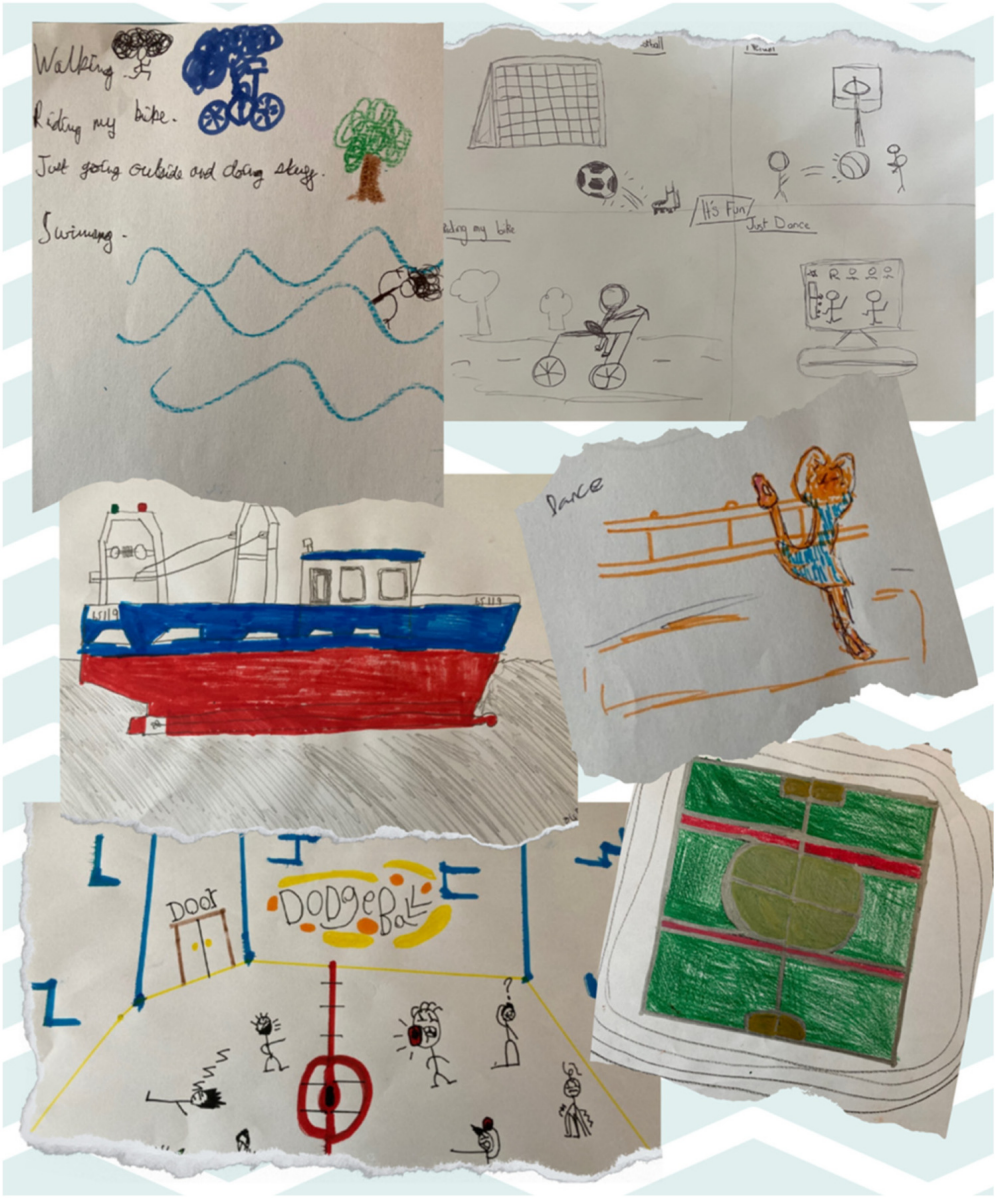
Example drawings from the participants showing meaningful activities involving moving one’s body.

When children were asked about activities they find meaningful that involve moving their bodies, they described a broad range of activities. These included traditional team sports, such as football and rugby, which are deeply embedded in British culture and widely promoted through school curricula and community programmes. However, children also frequently mentioned activities such as walking and cycling, which, while not typically classified as organised sports, hold significant value in fostering daily movement and physical engagement. In addition, some children highlighted intriguing non-traditional activities, such as Brazilian jiu-jitsu, surfing, skiing, tree climbing, fishing, and even cooking with their parents. These activities deviate from the conventional sports landscape often associated with British children, reflecting unique personal, family, or environmental influences. These findings suggest a need to broaden the definition of physical activity in future research to encompass diverse and culturally nuanced forms of movement. Recognising these activities as meaningful expands the understanding of what motivates children to engage in physical activity and highlights the importance of considering non-traditional forms of movement when developing interventions to promote lifelong activity. Figure 1 presents a word cloud of the activities mentioned (with the most common activities in the larger font), and Figure 2 provides examples of the children's drawings.

Through discussing how the children felt and what helps them do these activities, we identified four themes that capture why these activities were meaningful for children: (i) positive emotional responses to movement, (ii) importance of relationships with family and peers, (iii) psychological wellbeing, and (iv) physical health.

#### Positive emotional responses to movement

Irrespective of the activity, all the participating children described the activities they had drawn as emotionally rewarding. They said they felt “happy” (P14, P29) and described activities as “fun” (P4, P7, P13). One child expressed a sense of sadness when they had to stop playing football: “I kind of feel a little bit sad because I want it to keep going on and on.” Because “I liked it so much” (P2). Other children described positive emotions after taking part in physical activity. For example

“After walking, being out, see some trees, nature, I feel happy after that too.” (P14)

Others described this as “feeling excited” (P1), with rushes of adrenaline. For example, P2 said: “Yeah, usually with like cricket and football, you get quite a rush of energy.” For activities that require dedication to developing the relevant physical capabilities, children described a sense of accomplishment and reward from taking part:

“Dance requires a lot of time, skill and it makes you feel rewarded after you’ve finished dancing.” (P8)

“Like if you get a new personal best. It’s good like you’ve accomplished.” (P36)

It should be noted that children who were categorised as active/sporty and played team sports or athletics, described competition as being interwoven with positive emotional responses. Winning a game or getting a personal best often results in feelings of happiness and joy, whereas losing can lead to feelings of sadness. This underscores the importance of emotional self-regulation.

“Not if you lose if you’re losing its sad but if you win, you’re like happy.” (P34)

“Well, you feel quite upset. Like, if you’ve done well in the game, then you know you’ve tried you’re best, so.” (P29)

#### Importance of relationships with families and peers

The importance of relationships with friends and family was apparent across all participants. When children touched on a movement-based activity they found meaningful, more often than not, they mentioned that their friends or family were involved.

“I like it because I do it with my grandad.” (P34)

“Sometimes I play with my stepdad. And sometimes, like, get my football and play with my friends sometimes.” (P4)

“Playing with my sister, playing with my cat, and going out with friends to walk, like walk.” (P13)

These associations with friends and family were often discussed with feelings of happiness, enjoyment, and engagement. For example, P18 said, “it makes me feel really happy because I’m playing with my friends.” One participant mentioned the importance of doing the activity with someone you like for enjoyment purposes:

“Because I wouldn’t want to do it with someone that I didn’t like because it would just suck the fun out of it. Take all the fun out of it I think. I don’t know but, yeah it would get rid of all the fun.” (P15)

Furthermore, taking part in different types of activities was a prime opportunity to meet new friends:

“Yeah, because when you go to a squad you meet like, loads of new friends, not quite a lot of people get into the academy so you know, you like to speak to people know, that you might normally speak to.” (P29)

Children talked about how friends and family can help you learn how to get involved in certain activities and provide words of encouragement for one another:

“It’s nice to be with other people. So like you can sort of, like learn from like the people who’ve been doing it for like basically their whole lives.” (P32)

“What helps me ride my bike? My Dad. He like encourages me to do it, because when I first did it, I was like, I had to like have stabilisers but now I can ride without it because my dad encouraged me to go more.” (P31)

In some instances, children talked about their enjoyment from an activity because of a deeper meaning associated with their family. For example, P45 talked about the fishing being meaningful to them: “Because my father's a fisherman, and I’ve always grown up all my life around always going round fishing nets on a Sunday, that sort of thing.” Children described looking up to their parents in some instances and getting involved in a new activity because their family also played the sport. For example, P30 played rugby because her dad and brother did. These different activities then became more meaningful to the participants:

“I’ve drawn me and my Mum cooking together because obviously that involves moving around the kitchen, getting all the stuff … and it’s very important to me and means a lot to me.” (P42)

#### Psychological wellbeing

Children repeatedly talked about the psychological wellbeing benefits of taking part in meaningful movement-based activities. These emotions during and after physical activity included feeling “free” (P26, P53), “energetic” (P53), calming, clearing your mind, and letting go of any anger.

“Riding your bike is, it is fun same as just dance, but I guess you can feel the wind in your in your hair and like you could say you’re free from whatever, like school.” (P23)

“I put rugby because it just makes me feel happy. Because if I’m ever angry, then I can just take my anger out.” (P29)

“Because it clears your head.” (P48)

In addition, the participants reporting their “flow state,” in terms of getting lost in the moment through focus was also reported by several participants.

“When I do [football and boxing], I just get lost. I just get lost in it and I concentrate so much. I just lose everything around me.” (P2)

“You just like forget about everything else, so like when you’re in the game, you’re focusing on the game.” (P27)

These moments of concentration were linked to letting go of any worries or stresses and just focusing on the activity at hand. For example, P32 spoke about surfing as an activity that can help let “all your issues go away so you don't have to worry.” Some children specifically mentioned the benefits to their mental health:

“I feel a bit happier because it’s quite good for mental health and physical health, so if you’re feeling a bit stressed before, it can sometimes help you like, feel a bit better.” (P22)

There were some specific mentions of being outside having a positive impact on their mental health. P14 explained, saying, “see some trees, nature, I feel happy after that too.” In addition to happiness, there was a sense of serenity and appreciation for children talking about going for walks outside without any distractions.

“I really enjoy walking, well alone because when you go on a walk with multiple people, you’ve got to have a conversation going with them. And you don’t appreciate the scenery as much … But when you’re on your own, you appreciate the scenery around you a lot more. And I just really enjoy like just seeing things like my local area in a different way than I would with other people.” (P31)

#### Physical health

A small number of children mentioned taking part in physical activity and sports because of the health benefits. For example, P323 talked about feeling happy, knowing the physical activity was good for you: “happy for your body? I guess because like you’re exercising and it's good for your heart and lungs and stuff”. Similarly, P4 liked football “because it's good exercise” and P39 found boxing meaningful because “it keeps you fit and you’re working hard.” These insights highlighted the health-related motives behind engaging in movement-based activities.

### Moving your body for life, what's important? (task 2)

When children were asked about what might be important to move their bodies now and for the rest of their life, seven themes were identified: (i) knowledge of physical and mental health benefits, (ii) motivation, (iii) enjoyment, (iv) capability, (v) relationships, (vi) health and safety, and (vii) opportunity.

#### Theme 1: knowledge of physical and mental health benefits

Children reported a lot of physical and mental health benefits of being active during the life course, wanting to make sure you “stay healthy” (P2/P52) and “keep your health and wellbeing in the future” (P13). Many of the opinions were related to the physical health benefits of being active:

“It’s for your strength when you’re older and safe. Like, you can like you be stronger and healthy when you’re old.” (P4)

“This is more on your physical health. That if you move more, when you’re older, you will be less injury prone and, you’ll be like, more athletic, you won’t be like slow.” (P18)

Similarly, there were many descriptions around movement-based activities helping you “feel happy in life” (P19). Commonly, participants discussed the various benefits of being active on your mental health, especially as you get older and/or experience stressful events during your life:

“When you get older, your life gets a lot busier. So you need to do sport so you can relax your mind. And she said, you can be happy and don’t have to worry about anything else.” (P20)

“While doing it a lot of help here, it can improve mental health when stress, like in stressful situations.” (P31)

Most of the discussions with the children indicated that they knew the importance of being active and the encompassing benefits of being physically active.

#### Theme 2: motivation and physical self-regulation

Individual motivation was commonly reported by participants of all ages and activity levels as essential to being active and staying involved in their specific activities.

“I think that you need like to have lots of motivation.” (P15)

“Exercise can make you happier and improve your mental health, but if you’re already struggling with your mental health and you can’t find the motivation for exercise. Disabilities and injuries can prevent people from exercising … The area in which you live and your access to sports facilities can also make you struggle to find the physical activity that you enjoy so you won’t have the motivation to exercise more.” (P8)

Those less inclined to be active mentioned the importance of having an incentive to get involved, stating “a lot of sports … requires incentive, motivation” (P49). One participant said that buying “a new gym outfit, give me motivation,” which helped them feel “empowered” (P35).

Motivation was in some instances, associated with physical self-regulation and being goal-driven. For example, P38 said: “Yeah so, it's the motivation … Like you could say you don't like the way you look so the motivation to get in better shape. Just stick to it and grind the gym” (P38). There were further examples of carrying on with certain activities with the knowledge of the effort required and reasons to continue:

“You need, kind of the mindset to put effort into doing that as well because sometimes it takes a lot of effort to do some kind of sports.” (P53)

There was some discussion on the importance of establishing a healthy routine, with both diet and movement. For example, “I put down that you could do daily routines every single day” (P19) and “the amount of exercise you do every week, food that you may eat, just like have a routine” (P54). Other children went on to talk about the difficulties of breaking an unhealthy habit:

“I wrote that kids they should have actually be able to move and not just sit there. Because if you’re lazy, and … some people find it hard to move and it’ll be hard for them to come out of that habit.” (P11)

#### Theme 3: enjoyment

**Figure 3:**
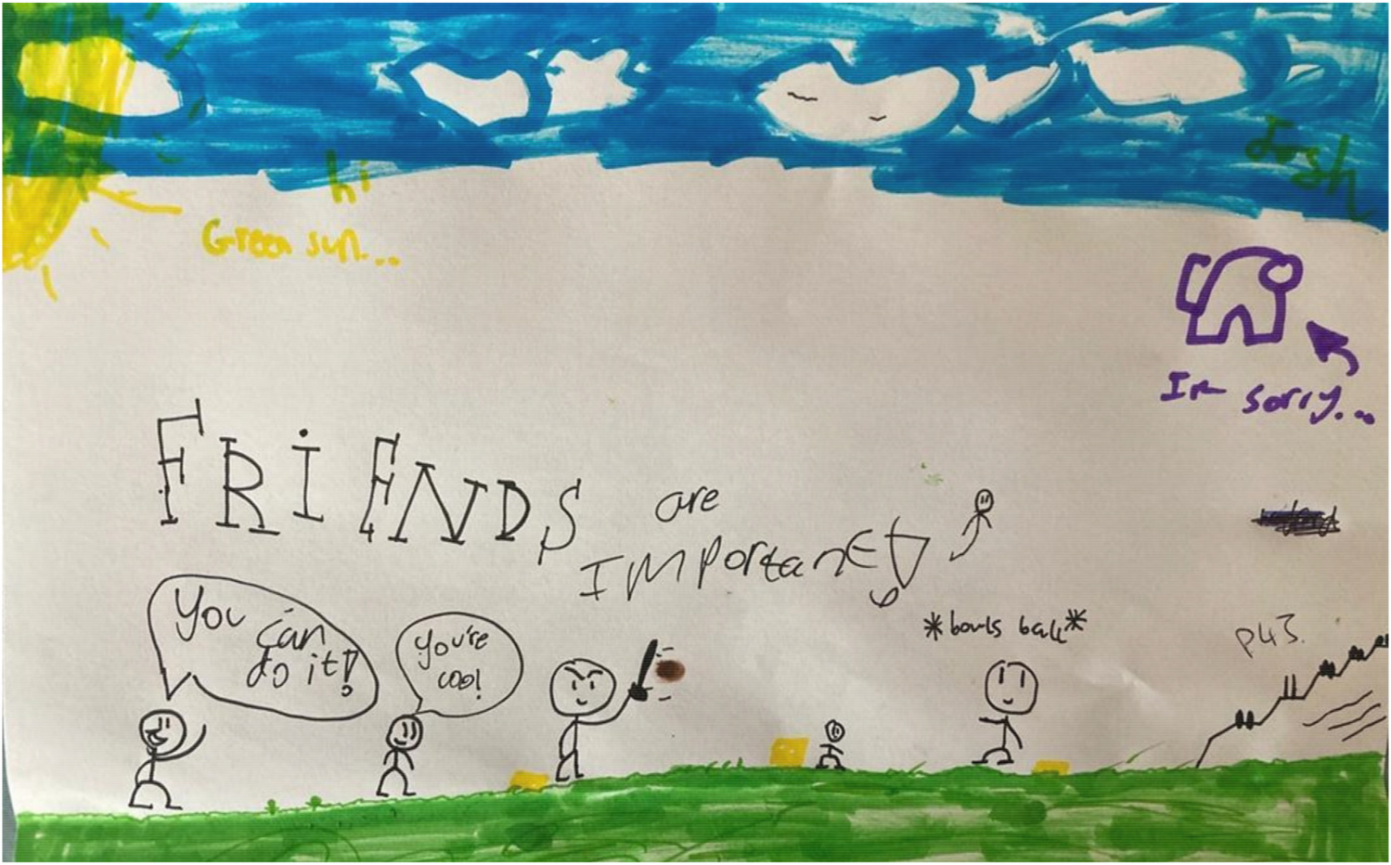
P43's drawing of the importance of friends.

Enjoyment during movement-based activities was seen as critical to ensuring consistency in engagement and motivation. For example, P34 mentioned that “if you don't enjoy it, then there's no point doing it.” Finding an activity that they find enjoyable and engaging was important for all the children, especially when considering doing these activities for the rest of their lives (Figure 3):

“I think in terms of physical activity, and doing it for the rest of your life, it’s important that you find enjoyment and you find the time and what works well for you.” (P27)

“I think enjoyment is important. Like if you enjoy what you’re doing, you’re going to do it for a long time and sometimes you can do it forever. So if you’re not enjoying something, you’re not going to do it.” (P14)

#### Theme 4: relationships

There was an emphasis on the importance of having friends and how they help increase your enjoyment levels. For example, P34 said: “I think it's important to do it with friends or with family. Because if you do it with people you don't know or people you don't like, you don't enjoy it as much.” There were also comments about the importance of friends in supporting you (even from afar), giving you confidence, making you strong, and encouraging you to be active, which helps enable you to get involved.

“If your friends encourage you to go more than if you go on your own.” (P36)

“Well, we have, you would have people to support you if things are not going well in sport. And it’s good to have like friends around you and support you that you’re doing as well, even if it’s by yourself because you might not have that many friends. Because some might go away to different countries.” (P53)

Following the discussion of the support of friends and family for encouragement, some of the KS3 active boys talked about having role models who helped them get involved in different activities and provided encouragement.

“I wrote football and I put next to it; I took over from my Dad because he got a serious injury.” (P1)

“Yeah. Because like for example, at first, like before I knew what football was, if I was with my dad, he can tell me how to play it. He like told me how to play it and what to do. And how, like the best way to get like goals.” (P3)

#### Theme 5: capability

Several children discussed needing to have the capability. P10 described that you need “capability and opportunity … not everyone can do exercise. So being there to do it. Having the opportunity and the chance.” This included physical capability, such as the “skills” of the activity and developing them over time. For example, P52 mentioned “just practicing the sport you want to do every day” can help you “improve your skills.” There was also a sense of the importance of a broader set of skills needed to take part:

“Like if you play sport, you’re going to have social skills and stuff … being able to work in a team and things like that.” (P13)

“You need to know about the sport as well. Because if the rules change or things change, then you’d be confused about it.” (P34)

In another example, P8 talked about enjoying netball because of the low effort needed, because they “knew all the rules but it's still fun to play,” suggesting that having knowledge of the rules and the understanding of how to play coincided with developing competence in the sport or physical activity.

Finally, some of the children mentioned that capability and specifically skill competence was seen as something “you can learn” (P38) and not something predisposed, leaning towards the notion that anyone is capable of engaging in movement-based activities.

#### Theme 6: health and safety

Children discussed the relevance of health and safety. This included looking after your health and trying to “reduce the risk of injury” (P52) by not overdoing it:

“I wouldn’t say to exercise or do sports every single day because you might get tired, and then you might get like on muscle pain or something … So exercise like three times a week, maybe two.” (P53)

There were also discussions about considering the physical environment around you and ensuring you have “a safe environment to do the sport you want to do.” (P17). Discussions then moved on to the safety and risks of sporting equipment, suggesting that while some equipment could be deemed dangerous, learning safety would alleviate these concerns:

“As long as you learn the safety, like hockey sticks are dangerous.” (P8)

#### Theme 7: opportunity

Having access to and the availability of different opportunities were regarded as important. For example, P35 mentioned they stopped dancing because of a lack of time and the cost of attending. Other participants mentioned the importance of having equipment, such as “having a bike” (P15) or having “toys” (P13). Some equipment-related needs can be alleviated: “Schools can provide equipment … to get [you] involved in different things” (P36).

P36 went on to state that when you leave school and lose access to and availability of the school-based opportunities. you might “have nowhere to go [yet] others join teams and clubs to carry on.” These discussions highlighted the importance of identifying potential opportunities that could encourage engagement in the relevant physical activities. One participant talked about country differences, stating that when they moved to England, the physical space and equipment to play hockey were significantly different compared to Zimbabwe, stating that: “I had access to the equipment … I played an actual match. But in England I didn't have access to that” (P8). P8 went on to state they stopped playing hockey soon after.

### PL elements (task 3)

The final task involved asking KS3 and KS4 children to rank the 30 elements defined by Barnett et al. and the Australian PL Framework (34) that underpin PL.

#### Important elements related to Pl for children in England

Of the 27 children who took part in this activity, the most common elements deemed meaningful and important were “motivation” and “confidence.” Almost synonymously, these two elements (in collaboration) were seen as critical to engaging in movement-based activity for the rest of your life.

“Because if you don’t enjoy it, and if you’re not confident to do it or motivated, then you won’t do it.” (P34)

“At the top, we put confidence because it’s not just something that comes naturally, it’s always like something that you got to build yourself. And there are always those people that tend to constantly think it’s their fault that they lost again because they don’t have too much confidence in themselves … Basically, confidence is like everything in everything. If you don’t have confidence in yourself, you can’t do it well.” (P31)

Additional common elements included perceptual awareness, i.e., being aware of your surroundings and “what's happening in the game” (P30). P37 felt awareness was of importance “Because, for example, if you’re in the gym, and you’re on the weight section, you’ve just got to be aware of [your surroundings].” (P37). This underscores the need for attentiveness in shared spaces where equipment or other participants might pose risks. Similarly, risks and safety were often reported as relatively important in most groups.

“You’ve got to put safety first. Not in everything. But if you’re riding a bike, or doing something that maybe not like classes, the safest, you’ve got to know that there is that possibility or chance that you will get hurt. You’ve got to know that …” (P28)

For some groups, connection to place was deemed relatively important. For example, P36 touched on motivation: “Connection to play. If you’re somewhere you like, you’ll be more motivated*.*” Conversely, some of the sporty/active participants felt this was not as critical.

“Connection to place like when you’re playing or where to go to like a difficult time you are connected to like you and pitch or different court or whatever sport you’re doing. So you’re connected to your home pitch it’s not really a big deal to play different teams.” (P29)

Society and culture were deemed important by most children, stating, “it's good to know about different cultures” (P17). At the end of the session, when the children reflected on the session and specifically task 3, there seemed to be a better understanding of the range of potential elements that are important to support moving your body for the rest of your life. P53 concluded by summing up its importance as “being socially active and being aware of your mental health as well.”

Almost consistently, children felt that the physical elements were not as important when compared to the other elements belonging to the social, psychological, and cognitive domains, with some groups completely disregarding these, whereas others felt particular attributes may be important. This was often subjective to the activities they had mentioned previously. For example, P30 and P29 felt object manipulation was important and they both reported playing rugby. During the discussions, there was a clear theme of children reflecting that most of the physical elements were dependent on the activity, and in most cases, they are not that important to help you engage in movement-based activities for the rest of your life.

“They don’t all like, apart from a few of them which you, like movement you need to be able to move to do all of them, but you know like not some of them it just depends on which sport you’re doing.” (P15)

## Discussion

The aim of this qualitative study was to gain a deeper understanding, from children’s perspectives, of what being active means to them and to inform the consensus statement for England on PL. The results of task 1 showed that the children indicated they find value in participating in sport and physical activity, describing positive emotions and both physical and psychological benefits. They also highlighted that being active with other people is important to them. Across both tasks, children consistently emphasised enjoyment, social connections, and an awareness of physical and psychological benefits as key to sustained activity. In addition, task 2 highlighted their awareness of long-term motivators such as capability and opportunity, particularly after school. In addition, they acknowledged that having the capability and opportunity to be active (in particular, once they had left school) and the motivation to continue would be important.

These findings resonate closely with the core elements described earlier, “physical, affective and cognitive learning” (14), in particular when the children were discussing what is important to lifelong activity (task 2). When discussing how they felt whilst doing activities meaningful to them now (task 1), the emphasis was very much on how they felt emotionally and psychologically. The elements of physical capability and physical benefits were deemed less prominent throughout the study. This contrasts with Young et al.’s (12) [and others e.g., Belton et al. (17)] observations that the physical domain is seen as dominant in discussions on PL (by adults). For these children, the “freedom” of thought and movement and the “enjoyment” and fun they experienced when participating with others were overriding.

The findings resonate with previous qualitative research on barriers and facilitators, as discussed in Martins et al.'s (11) review, however, the asset-based approach of asking children to discuss what was meaningful and what makes PA meaningful allowed them to focus on the positive contributions to their activity rather than what may prevent them. This provided insight into how children with varying activity levels define meaningful engagement, with some prioritising fun and social elements, while others valued personal achievement. Although this study did not impose a predefined PL framework, the emergent themes naturally align with key elements of PL, particularly within the affective and social domains. Children consistently highlighted the emotional aspects of activity, such as enjoyment, confidence, and motivation, which resonate with the affective domain. Similarly, their emphasis on social interaction and relationships strongly reflects the social domain, underscoring the importance of peer and family support in sustaining activity. It also reminds us that what children perceive as physical activity and how they conceptualise movement may vary from our preconceived ideas on what constitutes physical activity and why it may be important.

The importance of relationships to help you engage in meaningful movement-based activities now and for the rest of your life was continuously discussed during all three tasks. Taking part in a broad range of activities was often reported as enjoyable and encouraging when collaborating with others during these activities or having them there to support you and help you learn. The comments related to supporting and learning from each other not only reinforce the social domain but also suggest that physical competence is often developed through social engagement rather than in isolation. In some instances, the children mentioned enjoying activities on their own, for example, going for a walk alone, which offers different mental health benefits. However, more commonly, there was a direct association between social relationships supporting children's motivation. This suggests that any definition and consensus statement on PL should incorporate the social elements and how children and young people's affective responses to physical activity. This resonates with the findings of the Australian PL consensus process and definition, which found the social domain to be important alongside the other domains. Developing physical competence may be the first step, but being able to “work with peers, coaches, teachers and leadership” and transfer skills to a new environment are important for lifelong participation (18). Martins et al. (11) found social relations could be negative as well as positive, but friends were seen as fundamental to making activity enjoyable, providing psychosocial benefits and overcoming physical activity barriers, suggesting that developing social skills is also a fundamental aspect of PL. Again, this was a fundamental contribution to the work in defining PL and led to the inclusion of the social domain within the definition and consensus statement. These findings strengthen the wider literature due to our inclusion of more children from low socioeconomic and ethnic minority backgrounds. Our research also expanded beyond reasons for being physically active and explored what the important elements are for young people to be physically active now and for the rest of their lives. While this study intentionally allowed children's voices to shape the findings rather than fitting them into a predefined structure, the resulting insights reinforce the need to consider all dimensions of PL holistically. Task 2 required them to think about the future and what may help them be active; a longitudinal study following up on these anticipated barriers and facilitators may be beneficial. Whilst a study of this nature is not intended to be generalised to the population as a whole, the findings were similar across the range of socioeconomic and ethnic backgrounds and resonated with previous qualitative research, suggesting the findings are universal across backgrounds and cultures. These findings directly informed the development of the Physical Literacy Consensus Statement for England (10) by emphasising the need to incorporate affective and social dimensions alongside physical competence. Children's prioritisation of enjoyment, social interactions, and long-term motivation highlighted key elements that should be reflected in national frameworks. By capturing children's perspectives, this research highlighted elements of PL that are critical now and into the future for children. For the present, motivation and confidence were emphasised as immediate priorities, essential for fostering participation and enjoyment in physical activity. Looking forward, elements such as perceptual awareness and inclusivity were identified as foundational for creating sustainable and equitable frameworks that ensure long-term engagement. This layered understanding not only informed the English consensus statement but also provided actionable insights for tailoring PL initiatives to the evolving needs of children in England. By integrating physical, psychological, social, and cognitive dimensions, these findings offer a comprehensive approach to supporting children's overall wellbeing and their lifelong relationship with movement.

While this study provides valuable insights into children's perspectives on PL, we do recognise potential methodological limitations. One such consideration is social desirability bias, where the children may have shaped their responses based on what they perceived to be the “correct” or most acceptable answer while participating in focus group discussions (40). While the focus groups were designed to foster open and honest discussion, and the researchers felt this was achieved, it is possible that some children felt influenced by the presence of their peers, which is a common challenge in group-based research (40). The field researchers made concerted efforts to encourage all children to contribute, yet more confident or vocal participants may have inadvertently shaped group discourse, potentially limiting the diversity of perspectives captured. While individual interviews may have mitigated some of these effects, the focus group format was a deliberate methodological choice. It allowed for the exploration of socially constructed understandings of PL, providing valuable insights into how children collectively conceptualise meaningful movement experiences (41). The interactive nature of the group discussions revealed aspects of PL that may not have emerged in one-on-one settings, particularly in relation to the affective and social domains (40, 41). That said, a mixed-method approach, incorporating both individual interviews and focus groups, may be an avenue for future research, ensuring a balance between individual reflection and group dynamics. Another important consideration is the potential for variation in how children from different backgrounds and age groups perceive PL. While the findings were broadly consistent across participants, it is likely that younger and older children conceptualise PL in different ways (42, 43). Younger children may place greater emphasis on immediate enjoyment and social connection, while older children may develop a more nuanced understanding of physical competence and long-term benefits (42, 43). Socioeconomic and cultural factors could also play a role, influencing access to opportunities, perceived barriers, and the value placed on movement experiences. While this study did not aim to compare perspectives across these demographics explicitly, future research should examine these potential differences more systematically, particularly through comparative or longitudinal designs that track shifts in understanding over time. This study's findings did offer valuable insights into children's perspectives on PL, but we recognise the need for further research to explore how factors such as age, background, and context shape the development of PL over time. This should be a focus for future research.

## Conclusion

This study identified two key findings: first, children emphasised that immediate enjoyment, fun, and social interaction drive meaningful participation in physical activity. Second, children expressed the need for capability, opportunity, and supportive environments to sustain engagement in physical activity throughout life. These insights illustrate the necessity of building foundational skills, ensuring equitable access to diverse activities, and fostering an inclusive culture that promotes lifelong participation in movement-based activities. Future research should explore the long-term impact of PL-focused interventions on children's physical activity behaviours and overall wellbeing. Longitudinal studies could provide valuable insights into how motivation and confidence develop over time and their role in sustaining engagement. In addition, comparative studies across different cultural and socioeconomic contexts could help identify best practices for implementing inclusive PL programmes. Interventions should also consider the specific needs of underrepresented groups, such as children with disabilities or those from marginalised communities. Designing and evaluating programmes tailored to these populations will ensure that PL initiatives are equitable and accessible. Finally, greater emphasis on co-design approaches, where children actively contribute to the development of PL programmes, could enhance their relevance and effectiveness.

## Data Availability

The raw data supporting the conclusions of this article will be made available by the authors, without undue reservation.
