# Supplementary material for: Children's perceptions of physical literacy: exploring meaning, value, and capabilities for lifelong physical activity
Source: Front Sports Act Living. 2025 May 14;7:1548546. doi: 10.3389/fspor.2025.1548546 (PMC12116515; doi:10.3389/fspor.2025.1548546)
Supplement: Supplementary file 1 [file Table1.docx]

**TOPIC GUIDE *FOR Key Stage 2***

Date: Time:

School: Researcher(s):

Participants – please write first name in the table below.

| **“Active/Sporty/high PL”** | **“Inactive/non-Sporty/low PL”** |
| --- | --- |
| A1 – Male – WRITE NAME + Age + Ethnicity | I1 – Male – WRITE NAME + Age + Ethnicity |
|  |  |
|  |  |
|  |  |
|  |  |
|  |  |

**Introduction**

Hello everyone! My name is [*researcher name*], and I am here today to have a conversation with you all about what you think about activities you do while you MOVE YOUR BODIES - so this could be anything from walking, running, jumping, hopping, playing sports, doing PE, playing in the playground, riding your bike, swimming, and many other things. This could be things you do by yourself, but also things you do with family and friends in lots of different places (for example kicking a football in the park with your friend). This conversation is called a focus group.

The reason why we are doing a focus group is because people who work with children and young people across England, including researchers like me, want to find out what is important and what helps you do activities in which you MOVE YOUR BODIES. We all want to know so we can try and help all children and young people MOVE THEIR BODIES in any way they would like too now and when they are older. To do this we feel it’s important that we get your thoughts and opinions.

Your grown-ups/parents have given their permission for you to be here. This is not a test, so you are not being marked.

I will be recording what is said today [*show Dictaphone*] this is so we can record everything what we all say. Because we are recording, we need to take it in turns to speak, and not talk over each other. I will ask a question, then I will ask you what you think about it. If you would like to answer the question you can put your hand up, and we can take it turns to talk.

Let’s have a practice.

**Q. The first few questions are what is your name? And what is your favourite lesson in school, and why?**

I will go first my name is [*name*], and my favourite lesson in school was English, because I have always liked to read.

*[Go around the table for each child’s answer – use the space below to draw below where you and each child are sitting]*

|  |
| --- |

Ok now we have had a practice we all know what we are doing.

**Part 1 – Important activities**

**Q. In front of you are some paper and pencils and crayons [older children - clay, magazines etc]. I want you to spend 10 minutes drawing/crafting** an activity you find meaningful/important that involves moving your body.

*After 10 minutes*

Can everyone place your pieces of paper in front of you?

[If they ask you what you mean by meaningful ask them what is important to them – if you could do anything that involves moving your body you would choose to…]

Q. **Who would like to go first and tell the group what they have drawn?**

*[Try and avoid going around the table one by one, ask other children for their views, ask other children if they do the same activities, follow the below questions but bring in the wider discussion from the group.]*

- Prompt - Can you tell us a bit more about your activity?
- Prompt - What do you do when doing the activity?
- Prompt - How do you feel when doing the activity?
- Prompt - What would you say helps YOU do the activity?
- Prompt – How do you feel after the activity?
- Prompt – has anyone got something similar to XXX?

**Part 2a – The Key ingredients for someone to be active**

Carrying on from what you think helps you do your chosen activity. I now want you all to consider other children/young people your age and the different kinds of activities you and they do. So, think back of the different things we have been talking about already.

**Q. Can you individually spend 10 minutes writing down the things you think are important to move your body NOW and for the rest of your life. Don’t just think about yourself, think about other young people like you as well.**

- Prompt – What about their body, anything they need to do an activity?
- Prompt – What about their mind, anything they need to do an activity?
- Prompt – What about what they like?
- Prompt – What about who they spend time with when doing an activity?
- Prompt – What about where you might (physically) be?

**Q. Thinking about everything we have been discussing, what are the most important things that will help young people like yourself do activities that involves MOVING THEIR BODIES?**

Thank you so much for your time! We will let you and your teacher know what happens in the future. If you have any questions after today, please do ask your teacher to get in touch with me.

**TOPIC GUIDE *FOR Key Stage 3 & 4***

**Topic Guide – Key Stage 3 and 4 (young people aged 11 to 16)**

Date: Time:

School: Researcher(s):

Participants – please write first name in the table below.

| **“Active/Sporty/high PL”** | **“Inactive/non-Sporty/low PL”** |
| --- | --- |
| A1 – Male – WRITE NAME + Age + Ethnicity | I1 – Male – WRITE NAME + Age + Ethnicity |
|  |  |
|  |  |
|  |  |
|  |  |

**Questions are in bold**

**Introduction**

Hello everyone! My name is [*researcher name*], and I am here today to talk to you all about what activities you do while you MOVE YOUR BODIES - so this could be anything from walking, running, playing sports, doing PE, dancing, riding your bike, swimming, and many other things. This could be things you do by yourself, but also things you do with family and friends in lots of different places (for example kicking a football in the park with your friend).

The reason why we are doing this is because people who work with children and young people across England, including researchers like me, want to find out what is important and what helps you do activities in which you MOVE YOUR BODIES. We all want to know so we can try and help all children and young people MOVE THEIR BODIES in any way they would like too now and when they are older. To do this we feel it’s important that we get your thoughts and opinions.

Your adults (parents, careers) have given their permission for you to be here. This is not a test, so you are not being marked. What we are doing is something called a focus group, and it’s a way for us to explore and find out what you think about something.

I will be recording what is said today [*show Dictaphone*] this is so we can record everything what we all say. Because we are recording, we need to take it in turns to speak. I will ask a question then I will ask you what you think about it.

Let’s have a practice.

**Q. The first few questions are what is your name? and what is your favourite thing to do at the weekend, and why?** I will go first. My name is [*name*], and my favourite food to eat out is Italian because I love pizza.

*[Go around the table for each child’s answer – use the space below to draw below where you and each child are sitting]*

|  |
| --- |

Ok now we have had a practice we all know what we are doing.

**Part 1 – Important activities**

**Q. In front of you are some paper and pencils. I want you to spend 10 minutes writing (or drawing if prefer)** an activity you find meaningful/important that involves moving your body.

[If they ask you what you mean by meaningful ask them what is important to them – if you could do anything that involves moving your body you would choose to…]

**Q. Who would like to go first and tell the group what they have put down?**

*[Try and avoid going around the table one by one, ask other children for their views, ask other children if they do the same activities, follow the below questions but bring in the wider discussion from the group.]*

- Prompt - Can you tell us a bit more about your activity?
- Prompt - What do you do when doing the activity?
- Prompt - How do you feel when doing the activity?
- Prompt - What would you say helps YOU do the activity?
- Prompt – How do you feel after the activity?
- Prompt – has anyone got something similar to XXX?

**Part 2a – The Key ingredients for someone to be active**

Carrying on from what you think helps you do your chosen activity. I now want you all to consider other children/young people your age and the different kinds of activities you and they do. So, think back of the different things we have been talking about already.

**Q. Can you individually spend 10 minutes writing down the things you think are important to move your body NOW and for the rest of your life. Don’t just think about yourself, think about other young people like you as well.**

- Prompt – What about their body, anything they need to do an activity?
- Prompt – What about their mind, anything they need to do an activity?
- Prompt – What about what they like?
- Prompt – What about who they spend time with when doing an activity?
- Prompt – What about where you might (physically) be?

**Q. Thinking about everything we have been discussing, what are the most important things that will help young people like yourself do activities that involves MOVING THEIR BODIES?**

**Part 2b**

We have discussed a lot of different things and it’s all very interesting. Researchers from around the world have come up with 30 possible different things they think are important for young people to be do activities in which you move your bodies now but also for the rest of your lives.

**I am going to hand out slips of the 30 things. I want you together (pairs/groups) to go through and identify which activities you think are the most important.**

Go through the activities - use handouts or slides, give examples.

- - Prompt – Do you agree with any of the physical things?
    - Which ones are most important?
    - Which ones are not as important?
  - Prompt – Do you agree with any of the mental/psychological things? important?
    - Which ones are most important?
    - Which ones are not as important?
  - Prompt – Do you agree with any of the social things?
    - Which ones are most important?
    - Which ones are not as important?
  - Prompt – Do you agree with any of the thinking/cognitive things?
    - Which ones are most important?
    - Which ones are not as important?

**Is there anything missing here?**

**The final question is to ask you whether there is anything we have not discussed?**

Thank you so much for your time! We will let you and your teacher know what happens in the future. If you have any questions after today, please do ask your teacher to get in touch with me.
